# Impact of Increased Astrocyte Expression of IL-6, CCL2 or CXCL10 in Transgenic Mice on Hippocampal Synaptic Function

**DOI:** 10.3390/brainsci6020019

**Published:** 2016-06-17

**Authors:** Donna L. Gruol

**Affiliations:** Molecular and Cellular Neuroscience Department, The Scripps Research Institute, La Jolla, CA 92037, USA; gruol@scripps.edu; Tel.: +1-858-784-7060; Fax: +1-858-784-7393

**Keywords:** pyramidal neurons, Schaffer collaterals, LTP, neuroimmune, alcohol, field potential recordings, cytokine, chemokine

## Abstract

An important aspect of CNS disease and injury is the elevated expression of neuroimmune factors. These factors are thought to contribute to processes ranging from recovery and repair to pathology. The complexity of the CNS and the multitude of neuroimmune factors that are expressed in the CNS during disease and injury is a challenge to an understanding of the consequences of the elevated expression relative to CNS function. One approach to address this issue is the use of transgenic mice that express elevated levels of a specific neuroimmune factor in the CNS by a cell type that normally produces it. This approach can provide basic information about the actions of specific neuroimmune factors and can contribute to an understanding of more complex conditions when multiple neuroimmune factors are expressed. This review summarizes studies using transgenic mice that express elevated levels of IL-6, CCL2 or CXCL10 through increased astrocyte expression. The studies focus on the effects of these neuroimmune factors on synaptic function at the Schaffer collateral to CA1 pyramidal neuron synapse of the hippocampus, a brain region that plays a key role in cognitive function.

## 1. Introduction

Several lines of evidence have confirmed the existence of a neuroimmune system in the CNS, and a role for neuroimmune communication in CNS homeostasis, function, and pathology. Glial cells, and in particular astrocytes and microglia, are the main cellular components of the CNS neuroimmune system. Glial cells initiate neuroimmune communication primarily through the production of small protein signaling factors with distinct structure and function. These neuroimmune factors include members of the cytokine superfamily such as proinflammatory cytokines and chemokines. Typically, proinflammatory cytokines and chemokines are present at low levels in the normal CNS, while elevate levels are associated with CNS disease and injury. For example, elevated levels of proinflammatory cytokines and/or chemokines in the CNS are typical hallmarks of CNS inflammatory and neurodegenerative diseases such as HIV infection [1], Alzheimer’s disease [2], epilepsy [3], multiple sclerosis [4], alcoholism and fetal alcohol spectrum disorders [5,6,7], and psychiatric disorders (e.g., autism spectrum disorders, schizophrenia, depression) [8,9,10]. The elevated levels are thought contribute to pathological processes occurring in these conditions, although protective actions could also play a role. Elevated levels of these neuroimmune factors also occur in normal aging, and may play a role in cognitive decline that can occur with normal aging [11,12].

CNS glial cells are capable of producing a variety of proinflammatory cytokines and chemokines, but the specific biological actions and roles of these neuroimmune factors have yet to be fully elucidated, and are likely to depend on the cell source and physiological or pathological context. During conditions associated with CNS disease and injury, multiple neuroimmune factors are commonly, and often chronically produced. The complexity of this situation makes it difficult to identify the actions of specific neuroimmune factors and the cell source, especially if pharmacological, biological, or other types of tools are lacking. A number of approaches have been used to circumvent this problem. This article focuses on one approach, the use of transgenic mice that endogenously produce elevated levels of a specific neuroimmune factor in the CNS by a cell type that normally produces it, and within the anatomical integrity and physiological pathways of the CNS. The transgenic mice of interest in this review express elevated levels of the proinflammatory cytokine Interleukin-6 (IL-6), the chemokine CCL2 (CC chemokine ligand 2, previously known as monocyte chemoattractant protein-1 or MCP-1), or the chemokine CXCL10 (previously known as interferon-gamma inducible protein 10 or IP10) through increased astrocyte expression. The review summarizes studies on the consequences of the increased astrocyte expression on a basic mechanism of CNS function, synaptic function, and in particular, hippocampal synaptic function. The hippocampus plays a critical role in learning and memory, and alterations in hippocampal synaptic function can significantly affect cognition [13]. Studies in experimental models have shown that altered hippocampal synaptic function is associated with CNS conditions known to involve elevated expression of neuroimmune factors (e.g., [14,15,16,17,18,19,20,21,22,23,24,25,26]). The transgenic mice have also been a useful model for a number of other types of studies related to CNS conditions during disease and injury, a topic that is not addressed in this review (e.g., [27,28,29,30,31,32,33,34]).

## 2. Astrocytes Are a Primary Source of Neuroimmune Factors in the CNS

Astrocytes are the most abundant cell type in the CNS and a key component of the neuroimmune system of the CNS [35]. Astrocytes play a variety of roles in the CNS, as regulators/mediators of normal physiology and responders to adverse conditions, such as those occurring during injury and infection, when astrocytes contribute to repair and recovery processes [36,37]. A large number of cytokines and chemokines are produced by astrocytes, including IL-6, CCL2, and CXCL10, but relatively little is known about the specific roles and biological actions of these factors under physiological or pathophysiological conditions when astrocytes are the initial cell source of these factors. Astrocytes are in close association with neurons and synapses, making them ideally positioned to influence neuronal circuit activity, which is essential for normal CNS function and is often compromised in CNS disorders [38,39]. In this review, studies on the consequence of elevated astrocyte expression IL-6, CCL2, or CXCL10 on synaptic function at the Schaffer collateral to CA1 pyramidal neuron synapse of the hippocampus are summarized. The Schaffer collateral to CA1 pyramidal neuron synapse is one of the most highly studied synapse in the CNS [40]. Output from the CA1 region provides important input to other brain regions and plays a key role in learning, memory, and other cognitive functions.

## 3. Signal Transduction Pathways

**Figure 1:**
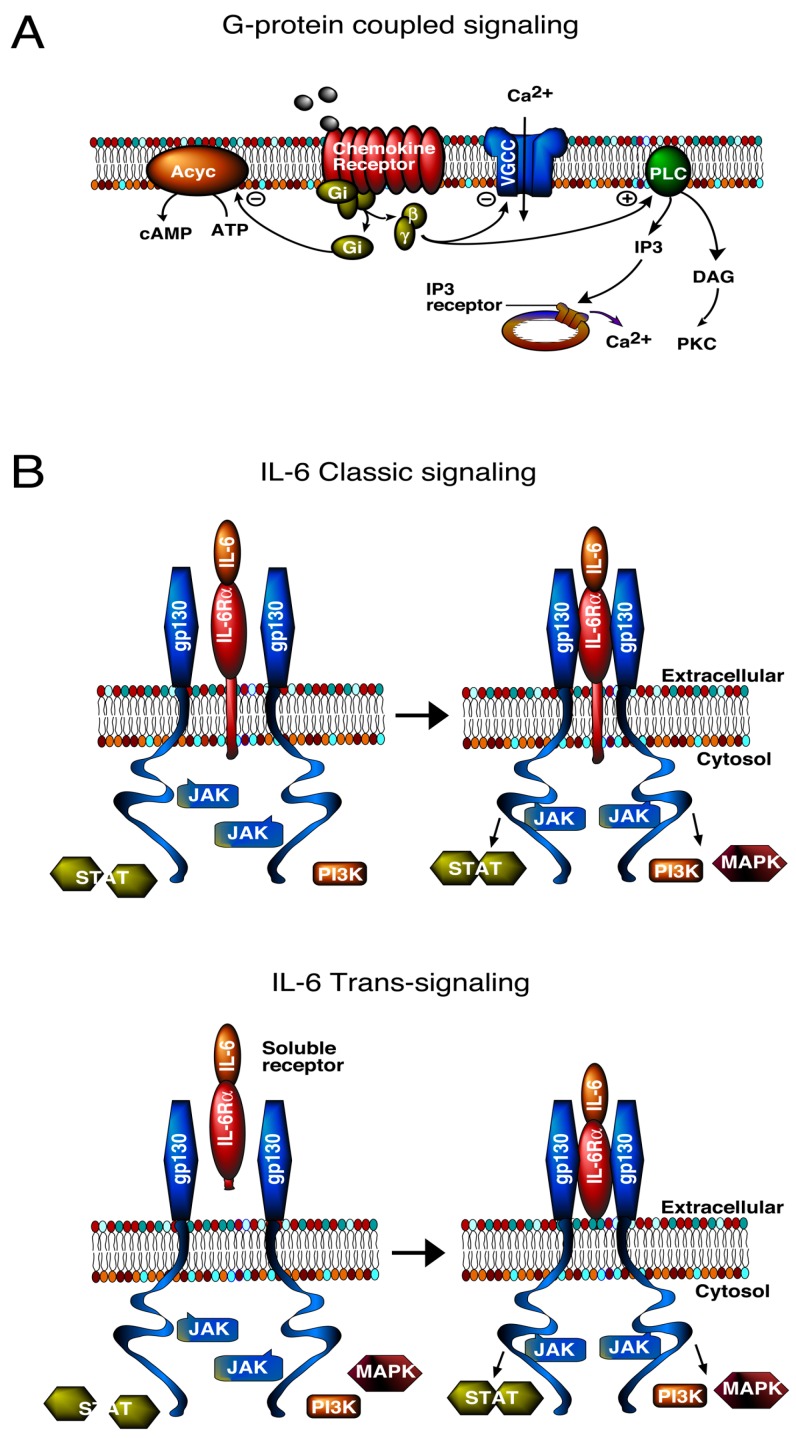
Diagrams showing signal transduction pathways used by chemokines and the proinflammatory cytokine IL-6. A plus sign within a circle indicates activation of the target molecule and a minus sign within a circle indicates inhibition of the target molecule. (**A**) Agonist binding to the G-protein coupled receptors (GPCR) initiates dissociation of the G-protein heterotrimer coupled to the receptor into Gα and Gβγ subunits. The Gα and Gβγ subunits then activate or inhibit downstream effectors. These effectors include ion channels, such as voltage-gated calcium channels (VGCC), and signal transduction molecules including phospholipase C (PLC) and adenylate cyclase (Acyc). Activation of PLC leads to the production of other signaling molecules including diacylglycerol (DAG) and inositol trisphosphate (IP3), and downstream activation of protein kinase C (PKC) and inositol trisphosphate receptors (IP3R), which regulate the release of calcium from intracellular stores; (**B**) IL-6 can signal through either a membrane bound (classic signaling) or a soluble (trans-signaling) IL-6R. The IL-6/IL-6R complex interacts with gp130 to activate the JAK/STAT signaling pathway. In addition, the IL-6/IL-6R/gp130 complex can activate RAS/mitogen-activated protein kinase (p44/42 MAPK, also called ERK1/2; MAPK) and phosphatidylinositol-3 kinase (PI3K) signaling pathways. All three signaling pathways activate additional downstream signaling molecules and effectors.

IL-6, CCL2 and CXCL10 initiate biological actions through the activation of specific membrane receptors, IL-6R, CCR2, and CXCR3, respectively. However, downstream signal transduction pathways differ. CCR2 and CXCR3 are G-protein coupled receptors (GPCRs), whereas IL-6R is linked to a tyrosine kinase signal transduction pathway (Figure 1). Moreover, IL-6R associated signal transduction can occur through two pathways, a classic pathway and trans-signaling [41] (Figure 1). 

The classic IL-6 pathway involves membrane bound IL-6R, which interacts with another membrane bound protein, gp130, the signaling subunit of IL-6R and other cytokine receptors. Trans-signaling involves IL-6R that has been released from cells into the extracellular fluid and is referred to as soluble IL-6R. Soluble IL-6R can bind to IL-6 in the extracellular fluid and the ligand/receptor complex can then bind to membrane bound gp130. Because gp130 is ubiquitously expressed in CNS cells, trans-signaling can occur in cells that do not express membrane bound IL-6R, and consequently trans-signaling greatly expands the target area of IL-6 actions. Trans-signaling appears to be the primary pathway involved in the pathological actions of IL-6 in the CNS [42]. 

The differences in signal transduction pathways utilized by IL-6 and chemokines could indicate different biological actions. However, signal transduction pathways downstream of the G-protein and tyrosine kinase step can merge at common pathway partners or targets and lead to similar biological actions. Thus, it is not surprising that all three neuroimmune factors have neuronal or synaptic actions, although the actions are not identical. 

Both neurons and glial cells express receptors and signal transduction pathways utilized by IL-6R [41,43], CCR2 [44,45,46], and CXCR3 [47,48], and are potential downstream cellular targets of the astrocyte produced neuroimmune factors. Because of the close association of astrocytes with neurons and synapses [39], actions of cytokines or chemokines on either cell type could potentially alter neuronal and synaptic function. Downstream molecular targets of GPCR and IL-6R pathways can regulate gene expression, which may be instrumental in directing neuroadaptive changes associated with elevated expression of IL-6, CCL2, and CXCL10 in the CNS of the transgenic mice.

## 4. IL-6, CCL2, or CXCL10 Transgenic Mice 

All three lines of transgenic mice with increased astrocyte expression of IL-6, CCL2, or CXCL10 were generated by a similar approach, insertion of the transgene (mouse or human) for the neuroimmune factor under transcriptional control of the glial fibrillary acidic protein (GFAP) gene promoter [29,34,49,50]. GFAP is an intermediate filament protein expressed almost exclusively by astrocytes in the adult CNS and commonly used as a marker for astrocytes [50,51]. More than one line was generated for each neuroimmune factor. Heterozygotes from the following lines were used for the studies discussed in this review: IL-6 transgenic line 167 (IL-6 tg), CXCL10 transgenic line CXCL10-10 (CXCL10 tg), CCL2 transgenic line on a SJL background (CCL2-tg SJL mice), and CCL2 transgenic line on a C57Bl/6J background (CCL2-tg), which were developed from the CCL2-tg SJL mice. Non-transgenic littermates of the respective transgenic line were used as controls. In general, elevated expression of other neuroimmune factors was not evident, or at low level in these transgenic lines [29,34,52], enabling investigation of the consequences of elevated expression of the transgene alone or in combination with other experimental manipulations. 

### 4.1. Expression of IL-6, CCL2, or CXCL10 in the Transgenic Mice

Because transgene expression in the transgenic mice is under control of the GFAP promoter, elevated expression of IL-6, CCL2, or CXCL10 is linked to GFAP expression. GFAP expression in astrocytes is initiated during the developmental period, which occurs primarily during the first 3 weeks of postnatal life in mice. GFAP expression in the mouse hippocampus is evident at 1 day postnatal, increases with age until 6 days postnatal, and then levels off and remains stable through adulthood [53]. Thus, neuronal/synaptic exposure to these neuroimmune factors in the transgenic mice occurs during an important period of structural and synaptic development and could affect developmental patterns. Evidence is limited on this topic, but in general, neuropathology in the hippocampus of the IL-6, CCL2, and CXCL10 heterozygous mice is absent or minimal up to 3–6 months of age, although homozygous mice can show pathology at early ages [29,32,54,55]. Thus, if the elevated expression of IL-6, CCL2, or CXCL10 altered CNS development in the transgenic mice, the effects on development were not pathological or were compensated for by other changes. In this review, discussion of the transgenic mice refers to the heterozygotes.

CNS expression of IL-6, CCL2, or CXCL10 has been quantified in the respective transgenic mice at the mRNA and/or protein levels. Studies of IL-6-tg mice showed that IL-6 mRNA was evident in the CNS at 7 days postnatal, increased with age and reached a peak at 3 months postnatal (adult stage), after which a decline was observed [52]. IL-6 transgene expression was demonstrated in hippocampal astrocytes by expression of the lacZ reporter gene and immunohistochemical detection of β-gal [55]. Constitutive secretion of IL-6 from astrocytes was demonstrated in studies of astrocyte cultures prepared from CNS of the IL-6 tg mice [49]. IL-6 levels were ~150 pg/mL in the supernatant from astrocyte cultures prepared from CNS of IL-6 tg mice, compared with <5 pg/mL for supernatant from astrocyte cultures prepared from CNS of non-tg mice. Interestingly, ELISA analysis of IL-6 levels in the hippocampus have revealed low levels and no differences between the IL-6 tg and non-tg hippocampus, although higher levels and genotypic differences were noted in the cerebellum [52,56]. The cerebellum is the CNS region with the highest level of IL-6 mRNA expression in the transgenic mice, particularly in the Bergman glial [49]. These results may indicate that IL-6 produced by hippocampal astrocytes *in vivo* is rapidly released and degraded. Others have noted difficulty in measuring IL-6 levels in CNS tissue using commercial ELISA kits, which may mean that there are technical issues to be resolved [57]. In spite of the lack of differences in measureable levels of IL-6 protein, increase expression of IL-6 regulated genes (e.g., GFAP, eb22, Socs3) and elevated levels of STAT3 and the activated form of STAT3 (phosphoSTAT3), the downstream partner of IL-6 signal transduction through which IL-6 acts to increase GFAP [58,59,60], were observed in the CNS of IL-6 tg mice. These results are consistent with actions of elevated levels of IL-6 in the IL-6 tg CNS. 

Protein measurements in the CNS of the two CCL2 transgenic lines showed that the older CCL2-tg SJL mice express higher levels of CCL2 in the hippocampus than in the CCL2-tg mice. CCL2 levels measured by ELISA were ~1.3 ng/mL at 3–4 months of age and ~3.0 ng/mL at 7–9 months of age in hippocampal homogenate from the CCL2-tg SJL mice [61]. In the CCL2-tg mice, CCL2 levels measured by ELISA were ~1.2 ng/mL at 3–5 months of age and ~1.5 ng/mL at 7–9 [58]. CCL2 levels were ~0.2 ng/mL in hippocampal homogenates from the non-tg mice from both the CCL2-tg and CCL2-tg SJL lines. Studies of supernatants from astrocyte cultures prepared from CNS of CCL2-tg SJL mice showed that astrocytes constitutively secrete large amounts of CCL2 (e.g., ~3.5 ng/mL) [34].

Expression of CXCL-10 in the CNS of CXCL10-tg mice has been characterized at the mRNA level by *in situ* hybridization [29]. The highest levels of CXCL10 mRNA were observed in the hippocampus, olfactory bulb, periventricular zone, cortical areas, cerebellum, and choroid plexus of the CXCL10-tg CNS (mice 5–6 months of age). Western blot studies confirmed high levels of CXCL10 protein in the hippocampus, and immunohistochemical staining confirmed expression of CXCL10 protein in astrocytes [29]. No CXCL10 mRNA or protein expression was observed in non-tg mice. Levels of CXCL10 protein in the CNS of CXCL10-tg mice have not been measured by ELISA. 

Elevated levels of neuroimmune factors are typically associated with pathological conditions, whereas low levels appear to exist under physiological conditions. However, the range of protein levels expressed during physiological and pathophysiological conditions has yet to be fully elucidated for most neuroimmune factors. Although elevated levels IL-6, CCL2 and CXCL-10 mRNA and/or protein have been documented in the CNS of the respective transgenic mice, it is unknown if protein levels for the three transgenic lines are functionally comparable. However, mRNA or protein levels were shown to be within the range associated with experimentally induced pathophysiological conditions in the CNS of IL-6 tg [62], CXCL10-tg [29] and CCL2-tg SJL mice [34]. 

### 4.2. Neuropathology

In general, before 3–6 months of age, the heterozygous IL-6, CCL2, and CXCL10 transgenic mice show relatively little neuropathology. In the IL-6 tg mice, the cerebellum shows the highest levels of IL-6 mRNA expression in the CNS of the IL-6 tg mice and greatest neuropathological changes, the most prominent being neovascularization [49,63]. Age-dependent neuropathological changes in the cortex and hippocampus of the IL-6 tg mice were evident in immunohistochemical studies of synaptic and cellular proteins. The neuropathological changes included reduced immunostaining for the presynaptic protein synapsin I indicative of synaptic damage (cortex, 12 months of age), reduced immunostaining for microtubule associated protein-2 (MAP-2) indicative of dendritic damage (cortex at 3 and 12 months of age), reduced immunostaining for parvalbumin, a calcium binding protein expressed by inhibitory interneurons (hippocampus at 3 and 12 months of age), and eventual loss of the interneurons, and reduced immunostaining for calbindin, a calcium binding protein expressed by inhibitory interneurons (cortex, 12 months of age) [49,54].

Histological studies of the CNS of CCL2-tg and CXCL10-tg mice are limited. However, CCL2-tg SJL mice have been reported to be free of neurological impairment before 6 month of age [34]. Routine histological analysis of the CNS of the CXCL10 mice showed no apparent neuropathological changes relative to the CNS of the non-tg mice [29].

## 5. Synaptic Function in the Hippocampus from IL-6, CCL2, and CXCL10 Transgenic Mice

**Figure 2:**
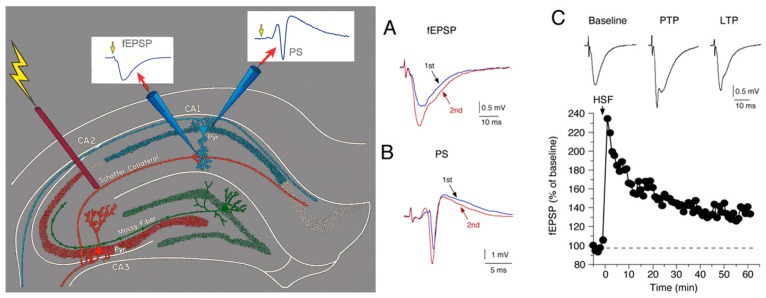
Measurement of synaptic function using extracellular recordings in hippocampal slices. (**Left Panel**) Simplified diagram showing the placement of stimulating and recording electrodes and recorded responses in a field potential recording of synaptic transmission at the Schaffer collateral to CA1 pyramidal neuron synapse in a hippocampal slice. Synaptic transmission is initiated experimentally by electrical stimulation of Schaffer collaterals, axons of the CA3 pyramidal neurons of the hippocampus. Stimulation of the Schaffer collateral elicits a fEPSP in the dendritic region and, depending on the strength of the stimulation, a PS in the somatic region; (**Right panel**) Repetitive stimulation can result in a change in the magnitude of synaptic responses. (**A**) Repetitive stimulation with a 40 ms interval between the first and second stimulation resulted in an enhancement of the fEPSP (2nd) evoked by the second stimulation relative to the fEPSP (1st) evoked by the first stimulation; (**B**) Repetitive stimulation with a 10 ms interval between the first and second stimulation resulted in an enhancement the PS (2nd) evoked by the second stimulation relative to the PS (1st) evoked by the first stimulation in this slice; (**C**) High frequency stimulation (HSF) induces a long-term enhancement of the fEPSP. The graph shows the magnitude of the fEPSP enhancement relative to baseline levels before high frequency stimulation was applied (at the arrow). The initial, large enhancement of the fEPSP is referred to as post-tetanic potentiation (PTP). The delayed, stable increase in the magnitude of the fEPSP is referred to as long-term potentiation (LTP). Representative recordings are shown above the graph.

For all three transgenic lines, physiological studies to assess synaptic function have been carried out at the Schaffer collateral to CA1 pyramidal neuron synapse of the hippocampus using a similar protocol that involved extracellular field potential recordings from acutely isolated slices of hippocampus (Figure 2). This approach has been extensively used for physiological studies of hippocampal synaptic function. One potential limitation to this approach is that the normal level of neuroimmune factors could be altered by the slice preparation and recording procedures. However, such effects would presumably also occur in the non-tg slices and thus be controlled for.

Synaptic transmission to CA1 pyramidal neurons was elicited by electrical stimulation of the Schaffer collaterals. Both baseline synaptic transmission elicited by single stimulations and synaptic plasticity elicited by repetitive stimulation were studied. The response to synaptic transmission was measured in the dendritic region of the CA1 neurons as a field excitatory postsynaptic potential (fEPSP), which reflects the membrane depolarization produced by synaptic transmission in a population of CA1 neurons (Figure 2). In some studies, recordings were also made in the somatic region of the CA1 pyramidal neurons, where population spikes (PS) were recorded (Figure 2). The PS reflects action potentials occurring in the soma/dendritic region that were generated by synaptic depolarizations in a population of CA1 pyramidal neurons. Data from hippocampal slices from the transgenic mice were compared to data from hippocampal slices from the respective non-tg littermate controls. Results are summarized in Table 1. In addition to the studies of IL-6 tg mice discussed in this review, two other studies of synaptic function in the hippocampus have appeared, both in the dentate region [64,65]. In addition, one study on synaptic function in the cerebellum has appeared [66].

### 5.1. Synaptic Transmission

The hippocampus from the IL-6 tg mice was studied at two ages, young mice 1–2 months of age and adult mice 3–6 months of age. Results were similar for the two age groups and showed that the fEPSP was enhanced in the hippocampus from the IL-6 tg mice compared to the hippocampus from non-tg mice of the same age group [60]. As a consequence of the enhanced fEPSP, the PS was also enhanced in the IL-6 tg hippocampus [60]. 

There was no difference in the fEPSP magnitude between the hippocampus from the CCL2-tg and non-tg mice at 2–3 months of age, whereas the PS was significantly larger in the hippocampus from the CCL2-tg mice [67]. Thus, the hippocampus from both the IL-6 tg and CCL2-tg mice showed an increase in the PS, indicative of increased excitability. However the increased PS in the hippocampus from the IL-6 tg mice could be explained by a larger fEPSP, but the increased PS in the hippocampus from the CCL2-tg mice could not. This difference indicates that although the functional consequence at the level of the PS was similar for the IL-6 tg and CCL2-tg hippocampus, different underlying mechanisms were involved. The increased excitability in the IL-6 tg mice could underlie the enhanced sensitivity to glutamate receptor agonists-induced seizure activity [69] and enhanced alcohol withdrawal hyperexcitability [70] observed in the IL-6 tg mice compared to the non-tg mice. The CCL2-tg mice did not show the enhanced alcohol withdrawal hyperexcitability observed in the IL-6 tg mice [70]. Effects glutamate receptor agonist on seizure activity has not been tested in the CCL2-tg mice.

In contrast to the CCL2-tg mice where only the PS was altered and an enhancement was observed, in the hippocampus from the CCL2-tg SJL mice at 7–12 months of age, both the fEPSP and PS showed a reduction in magnitude compared to non-tg hippocampus [61]. This difference between CCL2-tg and CCL2-tg SJL hippocampus may be due to the older age or the higher level of CCL2 expression in the CCL2-tg SJL hippocampus. In contrast to the IL-6 tg, CCL2-tg and CCL2-tg SJL hippocampus, there was no significant difference in the magnitude of the fEPSP or PS between the CXCL10 tg and non-tg hippocampus from 5–6 months old mice [68].

### 5.2. Synaptic Plasticity in IL-6 tg, CCL2-tg and CXCL-10 tg Mice

Synaptic plasticity is a change in the magnitude of synaptic responses that results when a synapse is repetitively stimulated. Synaptic plasticity is considered to be an important cellular mechanism of memory and learning [71]. Short-term and/or long-term synaptic plasticity at the Schaffer collateral to CA1 pyramidal neuron synapse has been studied in one or more of the transgenic lines. Results are summarized in Table 1.

#### 5.2.1. Short-Term Synaptic Plasticity

In this form of synaptic plasticity, repetitive activation of a synapse at short intervals (<1 s) elicits a transient increase or decrease in the magnitude of the synaptic response. Short-term synaptic plasticity is experimentally determined by applying repetitive stimulation to the Schaffer collaterals using a paired-pulse (P-P) paradigm. The magnitude of the plasticity is indicated by the paired-pulse ratio (PPR, magnitude of the response to the 2nd stimulation divided by magnitude of the response to the 1st stimulation). At the Schaffer collateral to CA1 pyramidal neuron synapse the paired-pulse protocol results in an enhancement the fEPSP (*i.e.*, a PPR greater than 1, Figure 2A). This enhancement is referred to as paired-pulse facilitation (PPF). PPF reflects greater transmitter release with the 2nd stimulation due to actions of residue Ca^2+^ on the probably of transmitter release in the presynaptic terminals of the Schaffer collaterals [72,73,74].

There was no difference in PPF of the fEPSP between the hippocampus from IL-6 tg and non-tg mice at either age studied (1–2 and 3–6 months of age) [60]. The hippocampus from CCL2-tg and non-tg mice studied at 2–3 months of age also showed no difference in PPF of the fEPSP [67]. In the 7–12 months CCL2-tg SJL mice, PPF of the fEPSP was increased at the 40 ms paired-pulse interval but not at longer intervals compared to the hippocampus from non-tg mice, indicating activity-induced presynaptic changes that impact excitatory synaptic transmission in a limited manner [61]. There was no significant difference in the PPF between the CXCL10 tg and non-tg hippocampus from 5–6 months old mice [68].

A second form of short-term plasticity induced by synaptic activation occurs in the somatic region of the CA1 neurons and affects the PS that is generated by the fEPSP. Plasticity of the PS can result in a PPR greater than one (less inhibition; Figure 2B) or less than one (more inhibition) depending on the relative contribution of somatic/dendritic excitability and recurrent inhibition to the somatic region. There was no difference in PPR of the PS between the hippocampus from IL-6 tg and non-tg mice at either age studied (1–2 and 3–5 months of age) [60], or between the hippocampus from CCL2-tg and non-tg mice at 2–3 months of age [67]. PPR of the PS in the hippocampus from 7–12 months old CCL2-tg SJL mice was increased compared to the hippocampus from non-tg mice, indicating decreased inhibitory influences in the soma/dendritic region [61]. There was no significant difference in PPR of the PS between the CXCL10 tg and non-tg hippocampus from 5–6 months old mice [68].

#### 5.2.2. Long-Term Synaptic Plasticity 

Long-lasting changes in synaptic transmission are also observed at the Schaffer collateral to CA1 pyramidal neuron synapse. These changes can involve an increase in the magnitude of the synaptic response, referred to as long-term potentiation (LTP), or a decrease in the magnitude of the synaptic response, referred to as long-term depression (LTD). LTP is experimentally induced by brief, high frequency stimulation of the Schaffer collaterals, whereas LTD is induced experimentally by prolonged stimulation of the Schaffer collaterals at low frequency. LTP has been studied in hippocampal slices from the IL-6 tg, CCL2-tg, CCL2-tg SJL and CXCL10-tg mice, but studies on LTD have not appeared. 

High frequency stimulation (HFS) of the Schaffer collaterals induces an immediate and dramatic increase in the amplitude of the fEPSP, after which the enhancement declines somewhat to a steady, stable level reflecting LTP (Figure 2C). The initial enhancement is a shorter form of synaptic plasticity referred to as post-tetanic potentiation (PTP). PTP results from the impact of HFS on presynaptic mechanisms involved in transmitter release. LTP, the delayed, persistent, stable increase in the magnitude of the fEPSP is primarily a result of activity-induced changes in post-synaptic mechanisms. Results from studies of long-term synaptic plasticity are shown in Table 1. 

In the IL-6 tg line, there was no genotypic difference in LTP between IL-6 and non-tg hippocampus from both young (1–2 months of age) and adult (3–6 months of age) mice. PTP was reduced in the hippocampus from young (1–2 months of age) IL-6 tg mice compared to the hippocampus from non-tg mice, indicating changes in presynaptic function, a genotypic effect that was not observed for IL-6 and non-tg hippocampus from adult mice (3–6 months of age) [60]. In both the CCL2-tg and CXCL10-tg lines, no genotypic effect on PTP or LTP was observed between the hippocampus from transgenic *vs.* non-tg mice [67,68]. PTP was enhanced in the hippocampus from the CCL2-tg SJL mice compared with the hippocampus from the non-tg mice, but there was no genotypic difference in LTP. 

### 5.3. Effect of Acute Application of Neuroimmune Factors on Synaptic Function

In addition to studies of the hippocampus from transgenic mice, studies on the effects of acute, exogenous applied IL-6 or CXCL10 on synaptic transmission and plasticity at the Schaffer collateral to CA1 pyramidal synapse have been carried out in hippocampal slices from rat or mice. The effect of acute exposure is of interest because it presumably reflects to some degree the actions of the endogenous cytokine or chemokine during the initial stages of elevated expression in the transgenic mice. Results are summarized in Table 2. There was no significant effect of exogenously applied IL-6 on the fEPSP or PS of rat hippocampal slices. There was also no significant effect of exogenously applied CXCL10 on the fEPSP in hippocampal slices from the CXCL10 tg and non-tg mice [68]. The effect of acute, exogenous applied CCL2 on synaptic transmission was studied in the rat hippocampal slices using whole cell voltage clamp techniques. CCL2 enhanced the excitatory postsynaptic currents elicited by stimulation of the Schaffer collaterals, an effect shown to result from actions of CCL2 on presynaptic mechanisms [75,76]. 

Although acute, exogenous application of IL-6 or CXCL10 had no effect on baseline synaptic transmission, IL-6 significantly reduced PTP and LTP in rat hippocampal slices [77,78]. Exogenous application of CXCL-10 also significantly reduced both PTP and LTP hippocampal slices from the non-tg mice, but only LTP in hippocampal slices from CXCL10-tg mice [68]. The lack of effect of CXCL-10 on PTP in the CXCL10-tg hippocampus, suggest neuroadaptive changes in the CXCL10-tg mice that prevent the actions of acute CXCL-10. 

Taken together, these results show that mechanisms that induce LTP and PTP are sensitive to acute exposure to IL-6 or CXCL10. Thus, the lack of genotypic differences in LTP and PTP between the IL-6 tg and non-tg hippocampus and the CXCL10-tg and non-tg hippocampus may reflect neuroadaptive changes in mechanisms that induce LTP and PTP. These neuroadaptive changes produced an apparent normalization of function. Effects of acute, exogenous application of CCL2 on PTP and LTP have not been reported.

## 6. Protein Levels in Hippocampus

IL-6, CCL2, and CXCL10 signal transduction pathways can lead to downstream effects on gene expression and, consequently, changes in the levels of important cellular and synaptic proteins. Changes in protein levels could also occur through other regulatory mechanisms. Such neuroadaptive changes could impact synaptic function. Western blot studies were carried out to identify potential changes in protein levels in the hippocampus from IL-6 tg and CCL2-tg mice. CXCL10-tg mice have not examined. Relatively few changes in protein levels were observed in the hippocampus from the IL-6 tg and CCL2-tg mice compared to hippocampus from their respective non-tg mice, as shown in Table 3. These results are consistent with the relative lack of neuropathological changes observed in the hippocampus from the IL-6 tg and CCL2-tg mice at the ages studied. However, some differences were observed that could affect synaptic function.

Compared to the hippocampus from non-tg mice, the hippocampus from IL-6 tg mice showed elevated levels of GFAP and STAT3, the signal transduction molecule that is involved in IL-6 regulation of GFAP gene expression [59,60]. The level of phosphorylated (*i.e*., activated) STAT3 was also elevated in the hippocampus from IL-6 tg mice [59,60]. Another astrocytic protein, glutamate synthetase, which is involved in glutamate cycling, an important aspect of excitatory synaptic transmission [80], was not altered in the hippocampus from the IL-6 tg mice [60], suggesting that the increased levels of GFAP do not reflect a general action of IL-6 on astrocytic protein levels. The increased levels of activated STAT3 in the hippocampus of IL-6 tg mice could affect synaptic function. STAT3 has been shown to be highly expressed in CNS neurons, where it is present in the postsynaptic density, and to regulate synaptic plasticity (LTD) in the hippocampus [1]. In addition to increased levels of GFAP and STAT3, reduced levels of GAD65/67, the synthetic enzyme for the inhibitory transmitter GABA, were observed in the IL-6 tg hippocampus [60], consistent with the immunohistochemical studies indicating a negative effect of the elevated levels of IL-6 on the structure of inhibitory interneurons [49,54]. 

Compared to the hippocampus from non-tg mice, the hippocampus from CCL2-tg mice showed elevated levels of synapsin 1, a presynaptic protein involved in transmitter release, and GluN1, the essential subunit of NMDA receptors [58,67]. NMDA receptors play a critical role in neuronal development, synaptic plasticity, and neuronal toxicity, and are an important target site for therapeutic intervention in a number of neurological disorders [81,82]. The neuroadaptive changes in synapsin 1 and GluN1 levels were not evident in the CCL2-tg SJL hippocampus, where the only changes were an increase in CD11b and GFAP [61]. Taken together, these results show that neuroadaptive changes occur at the level of synaptic proteins in the IL-6 tg and CCL2-tg hippocampus. The differences in proteins targeted in the IL-6 tg and CCL2-tg hippocampus could contribute to differences in the synaptic properties altered in the two transgenic lines.

## 7. Behavioral Studies

Alterations in synaptic function can result in changes in behavior. Two behavioral tests that evaluate the functioning of the hippocampus are the avoidance learning test and the contextual fear conditioning test. These behavioral tests were used examine hippocampal function in the IL-6 tg or CCL2-tg lines. Behavior has not been tested in the CXCL10-tg line. The IL-6 tg mice did not show a behavioral deficit compared to non-tg mice in the avoidance learning when tested at 3 months of age. However, by 6 months of age the IL-6 tg mice exhibited a significant deficit in their ability to learn the avoidance response, which declined further by 12 months of age [54]. The CCL2-tg mice were examined in behavioral tests for contextual fear conditioning at 2–3 months of age. There were no significant differences between the CCL2-tg and non-tg mice in these tests [67]. These results suggest the lack of significant hippocampal dysfunction at 3 months of age in both the IL-6 tg and CCL2 tg mice, at least under baseline conditions in these tests.

## 8. Covert Neuroadaptive Changes

Taken together, studies of synaptic function and protein expression in the hippocampus from IL-6 tg and CCL2-tg mice revealed relatively few neuroadaptive changes produced by the respective neuroimmune factor under baseline conditions, although the observed changes could significantly alter CNS function depending on physiological or pathological context. However, studies on the effects of acute alcohol on synaptic function in the hippocampus from IL-6 tg and CCL2-tg mice and their respective non-tg controls revealed covert neuroadaptive changes that resulted in an altered the response to alcohol (Table 4). For example, although there was no difference in the magnitude of PTP and LTP in hippocampal slices from IL-6 tg or CCL2-tg mice compared to their respective non-tg controls under baseline conditions, exposure to acute alcohol (60 mM) depressed PTP and LTP in hippocampal slices from non-tg mice from both the IL-6 and CCL-2 lines, while PTP and LTP in hippocampal slices from the IL-6 tg and CCL2-tg hippocampus were resistant to this effect of acute alcohol [67,70]. Thus, the hippocampus from the IL-6 tg and CCL2-tg mice showed a similar resistance to the depressing effects of alcohol on LTP and PTP. Differences in the response to alcohol were also observed between IL-6 tg and CCL2-tg mice in the effects of alcohol on the fEPSP and PS. For example, 60 mM acute alcohol reduced the fEPSP and PS in hippocampal slices from non-tg mice from the IL-6 and CCL2 lines and in hippocampal slices from CCL2-tg mice, whereas in hippocampal slices from IL-6 tg mice the same dose of alcohol increased the fEPSP and PS [67,70]. 60 mM alcohol is a pharmacologically relevant dose that would produce severe intoxication in humans.

A difference in response to alcohol was also observed in behavioral studies of alcohol actions. In one study of alcohol withdrawal hyperexcitability, IL-6 tg and CCL2-tg mice and their non-tg littermates were exposed to an acute, high dose of alcohol (4 gm/kg, i.p.), which initially causes sedation, but during the phase of declining blood alcohol levels, CNS hyperexcitability is produced. The hyperexcitability was measured by handling induced convulsions (HIC) [83,84]. The IL-6 tg mice showed significantly higher HIC scores than their non-tg controls, indicating greater hyperexcitability, whereas CCL2-tg and their non-tg mice showed similar HIC scores [70]. In behavioral tests for contextual fear conditioning, there were no significant differences between the CCL2-tg and non-tg mice under baseline conditions. Acute alcohol (1 gm/kg, i.p.) significantly impaired the non-tg mice but not the CCL2-tg mice in this behavioral test [67]. In contrast, in the rotorod test, which is considered primarily a cerebellar mediated behavior, CCL2-tg and non-tg mice show no difference in recovery from the effects of acute alcohol (2 gm/kg, i.p.) [67]. A similar result was obtained for the effects of acute alcohol (2 gm/kg, i.p.) on IL-6 tg and non-tg mice in the rotarod test (recovery time = 176.2 ± 9.3 min for non-tg and 171.2 ± 9.0 min for IL-6 tg).

Covert changes were also revealed in other studies of the IL-6 tg mice. Systemic exposure (i.p. injection) to a low dose of kainate or NMDA induced prominent seizures and lethality in IL-6-tg mice but not in the non-tg mice, which required a higher dose to produce such effects [69]. Also, basal plasma corticosterone levels were normal in IL-6-tg mice but, after restraint stress, abnormally increased levels were observed in the IL-6 tg mice compared to non-tg mice [85]. Thus, in addition to the detected neuroadaptive changes in baseline functions and behavior, covert neuroadaptive changes are produced by the chronic exposure to IL-6 and CCL2 and can be revealed within certain contexts. Such neuroadaptive changes could play an important role in pathophysiological conditions. 

## 9. Conclusions

Although a large literature has demonstrated elevated CNS expression of cytokines and chemokines in CNS disease and injury, a relatively small number of studies have examined the consequences of the elevated expression at the synaptic level. The transgenic approach provides tools for such studies. Transgenic models that target astrocyte production of neuroimmune factors have enabled studies that provide a basic understanding of the synaptic consequence of persistent elevated expression of a specific neuroimmune factor by this CNS cell type. This information can facilitate identification of potential contributions of the neuroimmune factor to a more complex condition when multiple neuroimmune factors are expressed. This information may also be useful for identification of the actions/role of specific neuroimmune factors in CNS physiology. The astrocyte targeted transgenic models complement traditional approaches involving knock out (KO) models. In the KO model, all cell types are affected and, therefore, the KO models provide more global information about the involvement of a specific neuroimmune factor in CNS development, function or dysfunction. One caveat to these models is that expression in the transgenic model or lack of expression in the KO model occurs over the lifespan of the animal, which could influence CNS development. It is unclear if or how potential development effects would impact studies in adult animals. However, emerging research on the actions of neuroimmune factors on CNS development is starting to provide answers to this question. 

Overall, the studies of synaptic function in the hippocampus from the three transgenic lines revealed relatively few alterations. This result is consistent with the relative lack of neuropathology in the hippocampus of the transgenic mice at the ages studied, and raises the possibility that additional factors may be necessary when pathology is observed. Both similarities and differences were observed in the effects of the three neuroimmune factors on synaptic function, suggesting that similarities and differences exist in underlying mechanisms, and are likely to be reflected in the consequences of elevated expression under different pathological contexts.

Although only a limited number of neuroadaptive changes in synaptic function were identified under basal conditions, several experimental manipulations revealed that covert neuroadaptive changes were produced by elevated expression of the neuroimmune factors. These covert neuroadaptive changes may have been responsible for the apparent normalization of function under baseline conditions such that genotypic differences were not observed. The identification of covert actions illustrates the importance of physiological or pathological context in the consequence of cytokine or chemokine actions in the CNS. Both the identified and covert neuroadaptive changes resulting from increased astrocyte production of the neuroimmune factors could contribute to cognitive impairment in a pathological context. 

The mechanisms and molecular targets underlying the neuroadaptive changes produced by IL-6, CCL2, and CXCL10 have yet to be elucidated. Studies to address these issues are an important future direction, and are essential for a more complete understanding of the actions and roles of IL-6, CCL2 and CXCL10 in CNS physiology and pathology. The level of expression, duration of exposure, presence of other neuroimmune factors, and biological context are all likely to be important variables, and their biological impact will also need to be resolved in future studies. Taken together, such information could reveal new targets for therapeutic intervention for a range of pathophysiological conditions that are associated with increased expression of IL-6, CCL2 and/or CXCL10 in the CNS. 

## Figures and Tables

**Table 1:** Genotypic differences in synaptic function in the hippocampus.

Measurement	IL-6 tg *vs*. Non-tg		CCL2-tg *vs.* Non-tg	CCL2-tg SJL *vs*. Non-tg	CXCL10-tg *vs.* Non-tg
Age (months)	1–2	3–6	2–3	7–12	5–6
Synaptic transmission					
-fEPSP	↑	↑	no Δ	↓	no Δ
-PS	↑	↑	↑	↓	no Δ
P-P synaptic plasticity					
-fEPSP (PPF)	no Δ	no Δ	no Δ	↑	no Δ
-PS (PPR)	no Δ	no Δ	no Δ	↑	no Δ
Long-term synaptic plasticity					
-PTP	↓	no Δ	no Δ	↑	no Δ
-LTP	no Δ	no Δ	no Δ	no Δ	no Δ
Reference	[60]		[67]	[61]	[68]

↓ = decrease, ↑ = increase, no Δ = no difference.

**Table 2:** Effects of exogenous application of neuroimmune factor on synaptic function in hippocampus.

Measurement	Neuroimmune Factor				
	IL-6		CCL2	CXCL10 non-tg	CXCL10 tg
species	rat	rat	rat	mouse	mouse
Age (months) or weight (gm)	2–3 months	200–250 gm	0.5–1 month	5–6 months	5–6 months
Concentration	1, 5, 50 ng/mL	50–2000 U/mL	2.3 nM	10 ng/mL	10 ng/mL
Synaptic transmission					
-fEPSP or EPSC	nd	no Δ	↑	no Δ	nd
-Population spike	no Δ	nd	nd	nd	nd
Short-term synaptic plasticity					
-fEPSP (PPF)	no Δ	nd	nd	↑	no Δ
-Population spike (PPR)	nd	nd	nd	nd	nd
Long-term synaptic plasticity					
-PTP	↓	↓	nd	↓	no Δ
-LTP	↓	↓	nd	↓	↓
Reference	[79]	[78]	[75]	[68]	

↓ = decrease, ↑ = increase, no Δ = no difference, nd = not determined. EPSC = excitatory postsynaptic current.

**Table 3:** Genotypic differences on protein levels in hippocampus.

Measurement	IL-6 tg *vs*. Non-tg		CCL2-tg *vs*. Non-tg		CCL2-tg SJL *vs*. Non-tg	
Age (months)	1–2	3–5	1–3	3–5	3–4	7–9
Housekeeping proteins						
-β-actin	no Δ	no Δ	no Δ	no Δ	no Δ	no Δ
Astrocyte proteins						
-GFAP	↑	↑	no Δ	no Δ	no Δ	↑
-Glutamine synthetase	no Δ	no Δ	no Δ	no Δ	nd	nd
Microglial protein						
-CD11b	nd	no Δ	no Δ	nd	↑	no Δ
Neuronal proteins						
-Enolase	no Δ	no Δ	no Δ	no Δ	no Δ	no Δ
-GAD65/67	no Δ	↓	no Δ	no Δ	no Δ	no Δ
Synaptic proteins						
-Synapsin 1	no Δ	no Δ	no Δ	↑	no Δ	no Δ
-VGLUT1	nd	no Δ	no Δ	nd	nd	nd
-GluA1	no Δ	no Δ	no Δ	no Δ	no Δ	no Δ
-GluN1	no Δ	no Δ	↑	↑	no Δ	no Δ
Signal transduction						
-STAT3	↑	↑	no Δ	nd	nd	nd
-p42/44 MAPK	no Δ	no Δ	no Δ	no Δ	nd	nd
Reference	[58,60]		[58,67]		[61]	

↓ = decrease, ↑ = increase, no Δ = no difference, nd = not determined.

**Table 4:** Effects of alcohol on synaptic function in hippocampus.

Measurement	60 mM Alcohol *vs.* Baseline			
	Non-tg	IL-6 tg	Non-tg	CCL2-tg
Synaptic transmission				
-fEPSP	↓	↑	↓	↓
-PS	↓	↑	↓	↓
P-P synaptic plasticity				
-fEPSP (PPF)	no Δ	no Δ	no Δ	no Δ
-PS (PPR)	↑	no Δ	↑	no Δ
Long-term synaptic plasticity				
-PTP	↓	no Δ	↓	no Δ
-LTP	↓	no Δ	↓	no Δ
Reference	[70]		[67]	

↓ = decrease, ↑ = increase, no Δ = no difference.
